# The ectopic pregnancy, a diagnostic and therapeutic challenge

**Published:** 2008-02-25

**Authors:** D Stucki, J Buss

**Affiliations:** Clinique de gynécologie–Obstétrique, Hôpital cantonal, Fribourg Swiss

## Abstract

The classic symptoms of ectopic pregnancy are secondary amenorrhoea, abdominal pain and vaginal haemorrhage, with a clinical picture of varying acuteness.

It is among the commonest causes of maternal mortality during the first three months of pregnancy

In the majority of cases (95%) the pregnancy is tubal, but other sites are possible (cervical, corneal, ovarian, peritoneal).

In the treatment of sterility or medically assisted reproduction, the risk of ectopic pregnancy should be borne in mind.

The individual risk factors may be cumulative, particularly with a previous history of extrauterine pregnancy or tubal surgery (including sterilisations), pelvic post–inflammatory status (adhesions proved by coelioscopy) or presence of an intrauterine device.

Diagnosis is based on serum beta–hCG concentration and transvaginal ultrasound

Laparoscopy is the treatment of choice for tubal pregnancies.

The decision to perform salpingotomy depends on the presence/status of a contra lateral tube.

In carefully selected cases local or intra–muscular administration of methotrexate allows conservative treatment, provided the patient does not present acute bleeding.

It is also indicated where trophoblastic tissue persists after surgery, notably salpingostomy, and in non–tubal ectopic pregnancies.

The latter are rare, however, and it is important to recognise them in view of the more serious complications.

## Epidemiology and risk factors

Ectopic pregnancy is not only a general cause of morbidity and mortality during recent years, but also a serious complication of pregnancy, responsible for 80% of maternal deaths that occur during the first trimester [1]. In combination with serum beta–hCG, its detection is made sooner and sooner due to the ultrasound image quality allowing the location of implantation site and therefore excluding an intracavitary situation. Most ectopic pregnancies are situated at tubal level (Table 1)

**Table 1:** Localisation of ectopic pregnancies

Tabl. 1: Localisation of ectopic pregnancies:
Tubal	
Ampullar	80%
isthmic	12%
infundibular	5%
Cornual	2%
Abdominal	1,4%
Ovarian	0,2%
Cervical	0,2%

At this moment, in many industrialised countries, the incidence of ectopic pregnancies is stable, even decreasing, due to the improved control of pelvic inflammatory disease.

The incidence of ectopic pregnancy increases with age: the risk of a 20 year old woman is 0.4% and grows between 1.3% and 2% at 30–40 years of age. This is probably linked to the fact that the chance of tubal lesions following inflammation of annexes or endometriosis grows with age. Also, the mean age of a planned first pregnancy went up in the last years in the industrialised countries. In case of sterility, frequently referred to medically assisted procreation techniques, the chance of developing an ectopic pregnancy is higher. A double intrauterine and extrauterine pregnancy was also described.

Other risk factors are linked to pelvic inflammatory diseases found through laparoscopy. A lot of studies carried out in the United States demonstrate that the high incidence of pregnancies in women of different origins is more due to the high percentage of pelvic inflammatory disease associated with a low socio–economical status than ethnical factors [2]. A history of tubal surgery (including sterilisations) represents equally a risk factor. The salpingotomy is a patent example. Women carrying sterilisation devices (hormonal or copper) have a higher risk of ectopic pregnancy. Smoking increases the chance of an ectopic pregnancy and depends on the number of cigarettes per day. The explanation takes into account the diminished tubal motility(Table 2).

**Table 2:** Risk Factors

Table 2: Risk Factors
History of tubal pregnancy
History of tubal surgery (and sterilisation)
History of pelvic inflammatory disease proven by laparoscopy
Implantable intrauterine device carrier
Endocrinology problems: dysfunctional ovulation, luteal insufficiency
Sterility treatment, assisted reproduction
Salpingitis isthmica nodosa
Nicotine

## Diagnosis

The diagnosis of extrauterine pregnancy is based on clinical examination, ultrasound and by following serum beta–hCG levels. Clinically, the ectopic pregnancy manifests by vaginal haemorrhage but also abdominal pain after a variable in duration secondary amenorrhea. Just as in the beginning of a normal pregnancy, the patient may have less specific symptoms such as nausea, vomiting and breast tension. Vaginal haemorrhage may be more or less abundant. They may be misinterpreted as being menstrual – especially when they appear after an interval of secondary amenorrhea, according to the patient's physiological cycle.

## Clinical Exam

Abdominal pain is caused by irritation of the peritoneum, through a more and more dilated tube or by the formation of a haemoperitoneum after retrograde blood flow coming from the tube or after tubal rupture. If the patient lies down on her back or in a Trendelenburg position because of haemorrhagic shock, blood may accumulate underneath the diaphragm and produce difficult to interpret pain. Differential diagnosis of low abdominal pain during pregnancy includes acute appendicitis, salpingitis, ovarian follicle or yellow body tear, ovarian torsion or a urinary pathways disease. 

## Complementary exams

The pregnancy is confirmed by beta–hCG detection in urine but also by quantity means in serum. Therefore, we commit to localise the pregnancy through transvaginal ultrasound. Generally, we admit that starting with a level of beta–hCG of 1500 UI/ml, we should visualise an intrauterine pregnancy, apart from a few exceptions like multiple pregnancies. It is important to point out that the diagnosis is not based on the absence of intrauterine image but on visualizing an image compatible with ectopic pregnancy, usually annexial [2]. In some cases where we do not see an exact location of the pregnancy, they are finally classified as ‘pregnancy with unknown location’, and only 10% prove to be real ectopic pregnancies. These patients must be followed through clinical exam, evolution of serum beta–hCG and especially by ultrasound search of implantation site. The ultrasound is certainly the most important exam that detects an extrauterine pregnancy with a sensitivity of 89% and a specificity of 99.8% [3].

**Fig 1:**
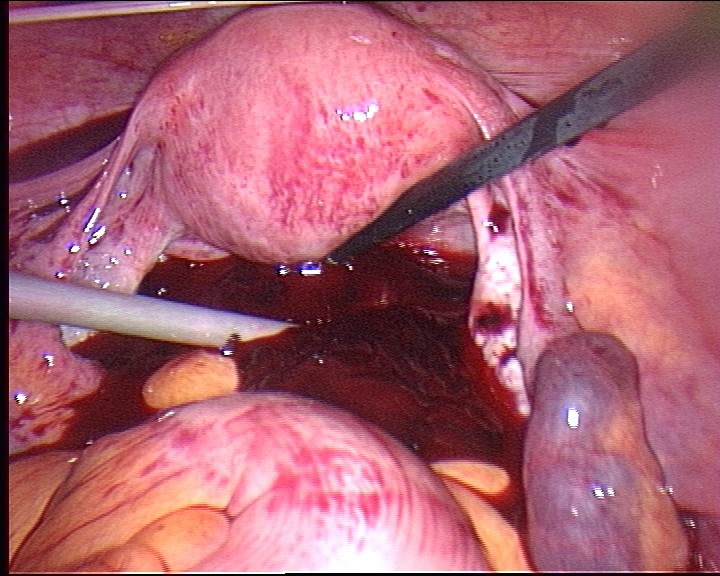
Laparoscopy site with haemoperitoneum in a right tube pregnancy

Ultrasound images are variable. The most frequent aspect is an annexial mass compatible with a gestational sack surrounded by a hyper–dense crown and a hypervascularity phenomenon during Doppler exam named as ‘chapel sign’ (Fig 1). The lesion can be equally visualised under the form of a heterogeneous solid mass. The detection of an embryonic structure, with or without cardiac activity is optional. The presence of yellow body must not be confounded with annexial pregnancy but this sign represents a good indicator for the location of an eventual extrauterine pregnancy which can be found in 85% of cases classified as ipsilateral. Generally and especially in symptomatic patients we find a clear fluid in the Douglas space corresponding to the accumulation of blood. In the uterine cavity, there is frequently described a ‘gestational pseudo-sack’ (Fig 3). Today, ultrasound equipments with high resolution do not allow us to distinguish between a true or false intrauterine gestational sack [4, 5].

## Treatment

Therapeutical option depends very much on the haemodynamic status of the patient and also on the location and age of the extrauterine pregnancy respectively.

### Surgical treatment

**Fig 2:**
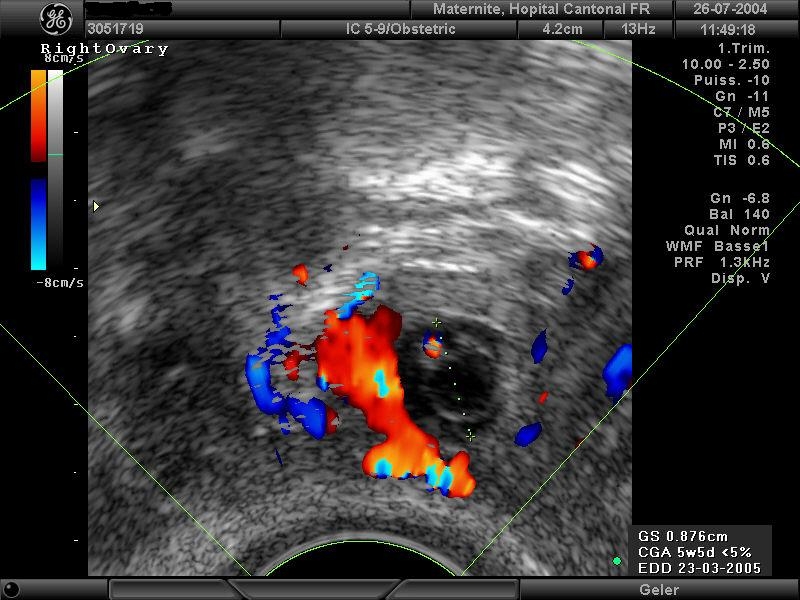
Right tubal pregnancy in the 6th week of gestation

In a symptomatic patient presenting clear free fluid in the Douglas space, an operative attitude is required. A laparotomy or a laparoscopy access depends very much of the haemodynamic status of the patient, on her surgical history and finally on the surgeon's experience especially in endoscopical procedures (Fig 2). Just as trials demonstrate that the approach does not influence the patient 's future fertility, celioscopic intervention has earned a value in terms of postoperative patient comfort. The laparotomy may, on the other hand be indicated for a haemodynamically unstable patient or presenting an important adherence status (history of infection or surgery).

### Salpingotomy or salpingectomy

In the case of a tubal pregnancy, two surgical options are possible: conservative treatment with salpingotomy followed by aspiration of tubal content and radical treatment with salpingectomy. It is obvious that the percentage of failure with the persistence of trophoblastic material is more important in salpingotomy (Fig 4). This will imply a higher risk of extrauterine ipislateral pregnancy. The discussion on whether which method is more appropriate depends on case to case. The question of future fertility remains open still because of the lack of randomised controlled trials. Success is associated not only with permeability of the concerned tube but equally with the status of the contra lateral tube. It was shown that fertility after salpingectomy in patients without any history of infertility with a normal contra lateral tube, comparable to that of those treated with salpingotomy in patients of less than 30 years of age. In case of ipislateral relapse, a salpingectomy is frequently necessary [6]. This is why, in cases without adherences, the remaining healthy tube will be capable of catching the ovule coming from the contra lateral ovary. In some rare cases, bilateral salpingectomy can be taken into consideration. For example, in the presence of a previous ipislateral extrauterine pregnancy and of an important contra lateral peri–annexial adherent status, this aggressive approach allows an increase in success percentage of medically assisted reproduction techniques. In the case when there is a will for sterilisation, it is obvious that a bilateral salpingectomy is equally indicated. After each conservative treatment of the tube, a serum beta–hCG based control is necessary.

### Medical treatment

Drug therapy with Methotrexate as first intent is reserved to stable, asymptomatic patients, without an evidence of haemoperitoneum on ultrasound or suspicion of tubal tear. As second intent, it is used in the persistence of serum beta–hCG percentage after surgical treatment by salpingotomy.

Several studies have shown that the success rate of primary treatment with Methotrexate reaches between 74–100% [5]. This rate depends greatly on serum beta–hCG levels: the less it is, the greater the chance for an ectopic pregnancy to completely disappear (Table 3). There are two ways to administer it: systemically (intramuscular) and locally, for example transvaginal. The last is particularly useful in the case of cervical pregnancy. The success percentage of 88% accompanies systemic application when beta–hCG is less than 5000UI/l and the pregnancy diameter is under 3 cm. A subgroup of patients with progesterone levels of more than 10ng can equally profit from an oral treatment of 600mg Mifepristone [8]. It is important to respect the contraindications of this product, like hepatic or renal diseases, bone marrow transplant, folic acid deficiency or presence of simultaneous intact intrauterine pregnancy. Two ways of intramuscular administration are currently used: either 50mg/m2, followed by a second injection if beta– hCG level doesn't decrease by 15% in the following 4–7 days, or 1mg/kg, days 1, 3 and 5 associated with folic acid in the second, fourth and sixth day [4, 5]. Economical benefit of such treatment fades if beta–hCG measurement must be repeated and if there is a need to secondarily refer to surgery for the persistence of the trophoblast [9].

**Table 3:** Success rates depending on beta–hCG level

Author	beta–hCG (U/l)	Success(%)
Condous	<5000	88
Sowter	<2000	97
	>2000	74
Tawfic	<4000	92,5
	>4000	35
Potter	<1000	98
	<5000	80
	>5000	38

It must not be forgotten that a third of pregnancies, independently of their location are spontaneously abortive. An expectative attitude though can not be adopted just like in asymptomatic patients and beta–hCG levels below 1500UI/l. In such cases, the rate of success reaches approximately 86%. They require though a clinical follow–up correlated with beta–hCG values in a very compliant patient [3].

### Non tubal pregnancies

About 5% of ectopic pregnancies are not located in the tube. This represents a minority but complications associated with other implantation sites are more serious, with haemorrhages and a rate of morbidity and mortality much higher.

Interstitial pregnancy (2%) can be seen by ultrasound in the form of a cornual ‘bulge’ located 1cm near the endometrium inside a uterine cone. The image may persist about 12 months after drug treatment.

Trophoblastic implantation at cervical level must be distinguished from an abortion product passing through the cervical canal (Fig 5). Different from cervical pregnancy, the false imminent birth is accompanied by pain. Doppler exam identifies the cervical implantation which is located underneath the entrance of uterine arteries. Such a diagnosis is important before the erosive lesion of uterine vessels will start a massive haemorrhage requiring surgical action by means of uterine artery embolisation or hysterectomy.

Ovarian pregnancy is les frequent (1/34). The ultrasonography image shows a hyperdense chorial ring which follows the movements of the ovary.

Abdominal pregnancy is a rarity, often diagnosed late in the presence of an empty uterine cavity and the unusual location of the foetus and of the placenta in the abdomen. Another rarity that we are noting is the post caesarean scar pregnancy. This pregnancy lies frequently in the retrovesical space having the risk of uterine rupture. Treatment of choice will be local or systemic application of Methotrexate [10].

**Fig 3:**
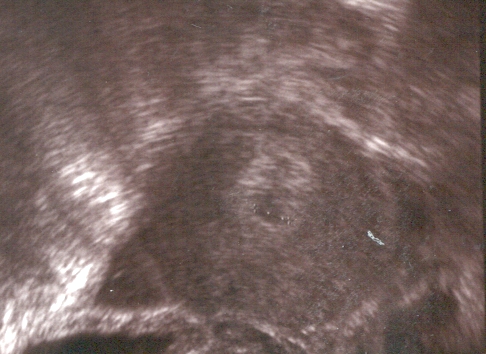
Accumulation of blood in the uterine cavity imitating a trophoblastic transformation(same patient as in images 4.1.–4.3.

**Fig 4:**
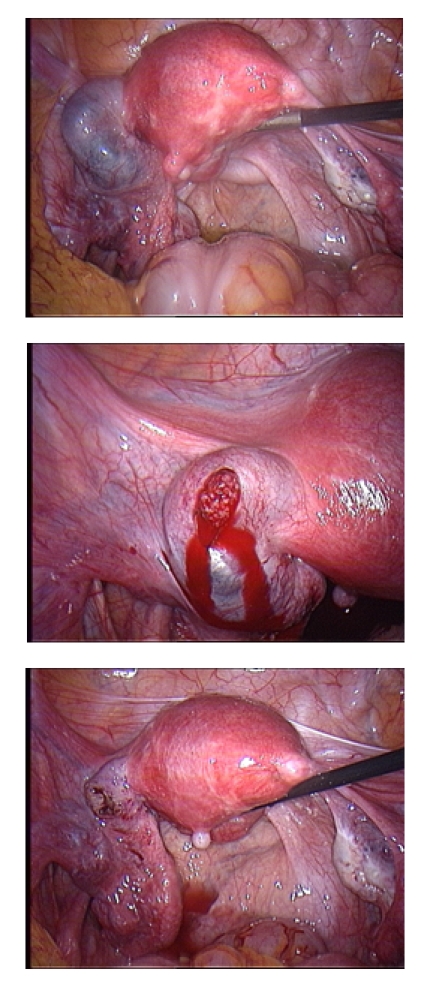
Left tube pregnancy (4.1.), after salpingotomy, we visualise the trophoblast (4.2.) which is completely extracted, incision remains open (4.3.)

**Fig 5:**
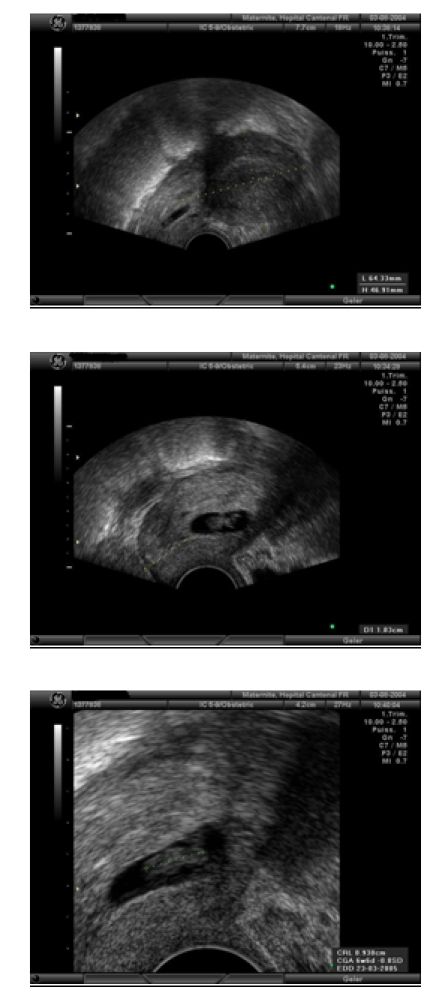
Cervical pregnancy in a 7 week of gestation patient with vaginal bleeding, 1.83cm away from the external orifice of cervix
